# Lectures on Subjects Connected with Clinical Medicine, Comprising Diseases of the Heart

**Published:** 1847-06

**Authors:** 


### PART II.—REVIEWS.

ARTICLE X.

*Lectures on Subjects connected with Clinical Medicine, comprising Diseases of the Heart.* By P. M. LATHAM, M. D., Fellow of the Royal College of Physicians, Physician Extraordinary to the Queen, and late Physician to St. Bartholomew's Hospital. Philad.: Ed. Barrington and Geo. D. Haswell. 1847. Being the October No., 1846, of the Select Medical Library. 8vo. pp. 365.

This work comprises thirty-eight lectures, and is replete with correct observation, clear illustrations of the pathology, and sound advice in reference to the treatment of the diseases of the heart.

That the object and plan of the work may be understood, we make the following extract from the author's preface:

"Mine is a limited purpose. It is to regard the diseases of the heart only in one point of view, *i. e.* as they appear in the living man. But this one point of view includes the several objects of their clinical diagnosis, and their medical treatment. These are what I seek especially to illustrate, while I presume an acquaintance with other parts of the subject, and shall only allude to them incidentally as I go along.

"The other parts of the subject, indeed, include no less than all that belongs to the morbid anatomy of the heart, the productions and processes which variously alter, or injure or destroy its organic structure. These are the very things of which clinical observation seeks to know the living signs, and the living history, and the treatment, both curative and palliative. But these, it is no part of clinical instruction formally to explain; yet, unless there be some previous knowledge of them, clinical experience cannot safely proceed; and unless that knowledge be kept up and improved concurrently with it, clinical experience can never go on to perfection.

"The clinical diagnosis of diseases of the heart owes all the higher degrees of certainty to which it has been carried in our own times, entirely to auscultatory signs. Accordingly it became necessary for me to give some account of their theory and their uses, and I have desired to do it as simply as possible. Their perfect theory, however, lies deeper than our present knowledge, and all the uses of which they are capable must wait to be developed by more and more multiplied observations of the sick.

"But already there is some true light in which these signs may be regarded; and already there is a large extent to which they may be followed and trusted as the faithful exponents of diseases of the heart. And although on this subject doubtless there has been error and mistake, and a good deal has been taken for more certain, and something for less certain, than it really is, so that we have both to learn and to unlearn; yet enough is already known to make the diagnosis of diseases of the heart hardly anything else than a just appreciation of their auscultatory signs.

"After I have described the auscultatory signs, and endeavored to show what they are, both in themselves and in relation to other symptoms, and what is their value as guides both to diagnosis and treatment, I may perhaps seem unreasonably to cut short the further description of the heart's diseases.

"But as I lectured, so now I write, for one class of students especially. As my hearers were, so now I presume my readers will be, chiefly those who are seeking information at the bed-side. To such there is no greater impediment of knowledge than over-teaching. The teaching which they most require is suggestive. They have the realities themselves to learn from, the original *book* to read, upon which all sound instruction is but a commentary. Therefore, the commentator should only interpose when and where he is needed, and not, after the manner of certain critics, who most *help* us with their annotations where the sense of the author is clear beyond dispute."

Lecture first treats of "the natural sounds, impulses, and resonances of the heart.—How their variations of degree and extent become evidences of the heart's disease or unsoundness."

That we may be able to enter upon a consideration of the subject understandingly, we make the following extracts from this lecture:

"Now it is evident that our inquiry must begin with the natural and healthy sounds and impulses of the heart. These are the standard of comparison, by which alone we can judge of the unnatural and morbid.

"First, then, of its sounds. And here, for the sake of avoiding confusion, let me just remark the distinction between the sounds which reach the ear simply by listening, and those which reach it by help of percussion. Though the ear judges of both, yet are they totally different in the modes of their production. *We* produce the latter, and the ear is made perceptive of them only by our knocking. The heart contributes nothing but as an inert mass; and what it contributes *as such* is found equally in the dead and in the living. It is the sounds which the heart brings out of itself, by its own vital movements, that I wish now to consider. The sounds which we bring out of the heart by our percussion I will consider hereafter: for they too carry with them notices of health and of disease, which are neither few nor unimportant.

"The sounds which naturally accompany the movements of the healthy heart, can only be learnt by the practice of listening to them. It is useless to describe them. They are simple perceptions of sense, which no words can make plainer than they are when the ear has once become familiar with them. It is the same with all common sounds. By describing them you seek to make them known in a different way from that in which they are naturally known. Who ever thought of describing the sound of the wind or the rain except for poetical purposes? I must leave you then to be your own self-instructors in the healthy sounds of the heart, and recommend you to be constantly practising auscultation for the purpose on healthy subjects.

"But, besides the fact, that sounds of a certain kind accompany the actions of the heart, which each man must listen for and so learn for himself, there is the theory of the fact, or the explanation how these sounds arise. This surely cannot be learnt by merely listening. The fact that it rains or blows, we may take upon ourselves to decide without the philosophers, because we hear it. But, if we would know how it comes to do either one or the other, if we would understand the theory of winds and showers, we must inquire a little further, and betake ourselves for instruction to those who have examined into such matters.

"In listening at the præcordial region, the ear at once perceives two sounds proceeding from the heart—the one duller and more prolonged, the other clearer and shorter; the one coinciding with the systole of the ventricles and the pulsations of the arteries, the other coinciding with the diastole of the ventricles and the rest of the arteries. Hence it appears that for one pulsation of the arteries there are two sounds of the heart.

"But between the two sounds of the heart there is hardly an appreciable interval. The duller sound, which goes for the first, seems to end with a snap, which goes for the second; and then succeeds an interval of repose, which is appreciable enough, before the duller sound returns.

"The time thus occupied by the sounds of the heart in their succession and their pause, has been divided and accounted for after this manner:—one half is filled up by the first sound, one quarter by the second, and one quarter by the pause."

These observations show how important it is to practice auscultation before anything can be relied upon in reference to the sounds of the heart by the pathologist.

After speaking of the sounds, resonances, and impulses of the heart in health, the author briefly states the variations from them in disease as follows:

"A clearer sound proceeds from a thin heart, and a duller sound from a thick heart; a sound of greater extent from a large heart, and a sound of less extent from a small heart. A more forcible impulse is given by a thick heart, and a feebler impulse by a thin one; the impulse is conveyed to a longer distance from a large heart, and to a shorter distance from a small heart.

"All this is surely plain enough, and it is undeniably true. Nevertheless, from its sounds *taken alone* and from its impulse *taken alone* we could come to few trustworthy conclusions respecting the structural condition of the heart. And why? Because its sounds and its impulses are capable of being augmented or lessened, both in degree and in extent, by causes extrinsic to the heart. This has been expressly stated already: and these extrinsic causes have oftentimes a power over its sounds and impulses as great as any which the heart itself derives from diseases of its own. This will be abundantly shown hereafter.

"But, happily, sounds and impulses are the interpreters of each other. The true meaning of the sound is tested by the impulse, and the true meaning of the impulse is tested by the sound.

"Thus, from a clearer sound, we argue only the probability of an attenuated heart; but we argue its certainty from a clearer sound joined with a weaker impulse. From a stronger impulse we argue only the probability of an hypertrophied heart; but we argue its certainty from a stronger impulse joined with a diminished sound.

"When impulse and sound increase together, there is probably no hypertrophy, but the heart is only acting more forcibly from pure excess of nervous energy. When impulse and sound decrease together, there is probably no atrophy, but the heart is only acting more feebly from pure defect of nervous energy.

"When the sounds and the impulse of the heart are both perceived beyond the præcordial region, they give notice, (generally speaking,) of dilatation of one or other of the ventricles. If, under these circumstances, sound predominate over impulse, then, with dilatation, there is either attenuation, or somewhat less than a proportionate increase of its muscular substance. If impulse predominate over sound, with dilatation there is either hypertrophy or somewhat more than a proportionate increase of its muscular substance."

Lecture second treats of *murmurs*, which are of equal importance with any of the symptoms emanating from the heart, as pathognomonic of its diseased conditions. All the unnatural sounds are termed murmurs; and are divided into the endocardial and exocardial:

"The endocardial murmur is not only different in kind from the natural sounds of the heart, but it takes their place and is heard in their stead. It comes exactly where the first sound, or where the second, or where both sounds should be. It keeps strict time with the systole or with the diastole of the heart, or with both.

"The exocardial murmur, too, is different in kind from the natural sounds of the heart. But it does not take the place of them. It is not heard in their stead. In proportion as it is louder, it obscures or overpowers the natural sounds. But the natural sounds are still apt to reach the ear through the exocardial murmur; and, when they do not reach the ear, it is because they are imperceptible under the circumstances, not because they cease to exist.

"It would be time and trouble thrown away to dwell long upon these endocardial and exocardial murmurs, with a view of describing what they are in themselves, and in contrast with each other. For after all every man must learn them for himself by the teaching of his own ear. Touching, however, our mere perception of them as sounds, there are a few circumstances interesting enough to mention which may chance to help the ear to a readier acquaintance with them.

"Whenever we hear any unusual sound either for the sake, of conveying our notion of what it is to another, or often, for the sake of being sure that we have a right notion of it ourselves, we are to set about imitating it. Now, any man hearing the endocardial murmur for the first time, as it occurs in the great majority of cases, would be almost sure to try and imitate with his *mouth*, and, what with whistling and blowing, he would presently hit upon something so very like it, as to make him pleased with his own cleverness. But hearing the exocardial murmur, such as it is in the majority of cases, for the first time, he would never think of imitating it with his mouth; he would rub his hands together or the cuffs of his coat, or take up any two things within his reach —two pieces of thick paper, perhaps—and rub them together and, what with brushing, and rustling, and crumpling, he would presently bring out a very near counterfeit of the exocardial murmur.

"But these murmurs are to be caught quickly and distinguished surely, and turned to a ready use, only by practice. Yet it gives a previous confidence in the reality of a distinction between them, to know that the endocardial murmur conveys to all ears the idea of blowing, and the exocardial murmur the idea of two bodies moving in contact with each other.

"It may be further stated among their general characteristics, that the endocardial murmur is most frequently a single sound, being coincident either with the systole or diastole of the heart; yet that sometimes it is a double sound, being coincident with both; but that the exocardial murmur is rarely less than a double sound. Moreover, that the endocardial murmur is commonly more inward and deeper, and further from the ear, and the exocardial murmur more outward and nearer to the surface, and closer to the ear."

In reference to distinguishing the valves of the heart that are diseased, by the position and direction of the endocardial murmur, he says:

* * * "The first fact is, that endocardial murmurs are most plainly audible at that part of the præcordial region which is nearest to the orifice from which they proceed. The second fact is, that endocardial murmurs are conveyed sometimes in one direction and sometimes in another, and that the orifice from which they proceed determines in each particular case what the direction shall be.

"Of these two general facts, I am more sure of the second than of the first, and have better proofs of its practical use. But we will briefly consider them both.

"A line drawn from the inferior margins of the third ribs across the sternum, passes through the pulmonic valves a little to the left of the mesial line and those of the aorta lie behind them, but about a half an inch lower down."*

* Hope on Diseases of the Heart, p. 3.

"'A horizontal line drawn through (along?) the under edge of the sterno-costal articulations of the fourth ribs will cut across nearly the middle of the length of the mitral valve, when drawn outwards and downwards by its tendinous chords and columnæ carnaæ, and pass about two or three lines above that portion of the tricuspid which most nearly approaches it, the latter valve lying underneath the sternum, and the former immediately to its left.'*

"So much of the sternum as these lines include to the left of the mesial line, and the space they indicate between the lower margin of the third and the lower margin of the fourth sterno-costal cartilages on the left side, may be taken to mark that portion of the præcordial region, behind which lie all the orifices of the heart, and a good share of the valvular structures appertaining to them. Now, inasmuch as the several orifices are found at the basis of their respective valves, the pulmonary and aortic orifices must be lower than the first horizontal line, and the tricuspid and mitral orifices must be higher than the second. How nearly, then, must they all approach one another in the mid-space between them both! So nearly that the mouth of an ordinary-sized stethoscope would surely cover them all within the circle of an inch and a half or less. Whichever orifice of the heart be affected, we are sure to find the endocardial murmur here or hereabout. And listening here and here only, we cannot segregate the murmur of one orifice from that of another. What then, if 'endocardial murmurs *are* most plainly audible in that part of the præcordial region which is nearest to the orifice from which they proceed!' This general fact, taken alone, cannot help us much in determinng which of them is affected in a particular case, when they all lie clustered together at the same, or nearly at the same, part of the præcordial region.

"But suppose we raise our ear or the stethoscope from this exact spot, and shift it an inch or two higner or an inch or two lower. Higher we may hear the endocardial murmur still, and lower we may lose it altogether. Or higher we may lose it altogether, and lower we may hear it still. Or both higher and lower we may still distinctly hear it. By this procedure we are following the endocardial murmur in the direction it takes after it leaves the orifice from which it is propagated, and we find how various the direction is, upwards in one case, downwards in another, and both upwards and downwards in a third. But still it is the orifice from which it is propagated, that gives the murmur its particular direction; and this, it is said, may be taken for a general fact."

* Joy, in Library of Medicine, vol. iii., p. 258, in a note.

"Among the conditions which may possibly intervene to turn endocardial murmurs from the direction in which the disease of particular valves would tend to convey them, the following may be mentioned:—

"1st. The presence within the chest, and exterior to the heart, of substances having a more solid consistence than its natural contents, such as morbid growths of various kinds, or aneurismal tumors, or condensed portions of lung. These are able to conduct the abnormal murmurs, no less than the natural sounds of the heart, to a greater distance, and in any direction, according to the place they occupy.

"2dly. The enlarged capacity of the heart itself, which is the most frequent consequence and concomitant of its diseased valves. The large dilated heart spreads its sounds abroad laterally. And thus, whether the murmur be traced in the course of the aorta, or not at all above the basis of the heart, it is often as loudly audible from mamma to mamma, and everywhere below the fourth ribs, as in the præcordial region itself; and often even far round towards the left axilla.

"3dly. The mere loudness of the endocardial murmur. The abnormal murmurs, as well as the natural sounds of the heart, are heard to a greater distance in proportion to their mere loudness, and that not only in the directions to which the current of the blood conducts them, but in all directions."

The subject is continued in lecture third, and the circumstances liable to interfere with or obscure the endocardial murmurs, as deformed chest, external pressure, murmur of respiration, especially in diseased conditions of the lungs, and from impoverished blood, are discussed.

The exocardial murmurs depend upon the friction between the surfaces of the heart and the pericardium when deprived of their natural smoothness by depositions from diseased action.

Lectures five, six, seven and eight, treat of inflammations of the heart under the heads of endocarditis and pericarditis:

"Endocarditis is at present chiefly known as a concomitant of acute rheumatism.

"In the year 1826 I was the first to teach the students of this hospital the fact that whenever the heart was affected in acute rheumatism, a sound different from the sound of health always accompanied its contraction. This was then a new fact, and one of immense importance; and all succeeding observation has gone to confirm its truth. This sound, in the vast majority of instances, was the bellows murmur. But in some, instead of the bellows murmur, it was some strange sound difficult to describe; and in others this strange indescribable sound and the bellows murmur seemed to occur together: there was a mixture of both.

"My notion *then* was, that all these sounds arose in some way or other out of inflammation of the pericardium; and taking them all severally and in combination, as the signs of pericarditis, I was amazed to find how far the frequency of its occurrence in acute rheumatism exceeded the common calculation and belief.

"In process of time I found, upon a comparison of cases, that where, in acute rheumatism, the bellows murmur occurred *alone*, the affection of the heart was upon the whole far less severe and far less perilous to life, than where some other unnatural sound occurred alone or in combination with it. I observed, too, that in some cases where the bellows murmur was unequivocal, the patient betrayed no uneasiness, no palpitation, in short, no other symptom which could give the least suspicion of a diseased heart; yet that in the great majority of instances where it once existed it remained permanent as long as the patient continued under my care and observation.

"At length I began to doubt whether the bellows murmur arising in the course of acute rheumatism was really derived from the pericardium, and to suspect that it proceeded from the internal lining. But for years the practice of this great hospital did not afford me a single opportunity of resolving my doubt, or of confirming my conjecture. For of that disease of the heart, which, coming on during acute rheumatism, is characterised by the bellows murmur, no patient of mine ever died, and I could learn nothing about it from dissection. But what my own experience would not furnish, M. Bouillaud's has supplied. Many have died during the active progress of this disease under his care, and dissection has found it to be inflammation of the endocardium. Thus we are indebted to M. Bouillaud for our first knowledge of this important fact.

"Nearly about the same time Dr. Watson and Dr. Stokes, of Dublin, further illustrated this important subject by separating *those other* sounds of the heart, which I have mentioned, to occur in acute rheumatism, from the bellows murmur, and analysing them apart. These they found to possess the character of attrition, as if produced by surfaces moving to and fro upon each other, and traced them home to their local origin in the pericardium, and showed the condition of their production to be a defective lubricity, or a ruggedness and unevenness of that membrane, such as would result from inflammation. In short, they showed these to be the proper signs of pericarditis, as the bellows murmur is of endocarditis; that when in acute rheumatism either occur alone (as it often does), the disease is simple pericarditis or simple endocarditis; and that when in acute rheumatism both occur together (as they often do), there is a mixture of the two diseases in the same subject.

"The bellows murmur coming on in the course of acute rheumatism is a sure sign of inflammation of the endocardium. Here, then, we will drop this exceedingly vulgar name, and call it "endocardial" again. But observe, it is the general character of the morbid actions predominant in the system at large which determines the particular character of the local disease out of which the endocardial murmur arises. *They* are inflammatory, and *it* is inflammation.

"In endocarditis, besides the endocardial murmur, there may be other symptoms present, directly referrible to the heart or there may not. There may or may not be pain. There may or may not be an excessive impulse, or an intermittent, irregular, or fluttering action of the heart. But the fact of endocarditis is not rendered more or less certain by their presence or absence.

"There may be doth pain and palpitation; yet endocarditis cannot be surely inferred to exist, unless there be the endocardial murmur withal.* There may be neither pain nor palpitation, yet endocarditis cannot be inferred to exist, if the endocardial murmur alone be present.

"Seeing, then, that the endocardial murmural one can determine the existence of endocarditis, you are required to search after it in every case of acute rheumatism. I say emphatically to *search after it*, because it is one of those signs which must always be sought before it can be found. It does not intrude itself upon our notice like palpitation or an irregular pulse. The patient does not draw our attention to it as he does to pain. The physician must make it out entirely for himself. And, indeed, it is infinitely important that he should have the earliest possible notice of it, with a view to the earliest possible application of the remedy.

"Never omit, therefore, to listen to the præcordial region whenever you visit a case of acute rheumatism, and visit a case of acute rheumatism oftener perhaps than you otherwise would do merely for the sake of listening. All may seem to be going on well. The general symptoms may be far from severe. The chest may be free from pain. The

* I do not say that it would not be fairly suspected, and that it would not be right to act as decidedly upon the suspicion in such a case as upon the matter of fact. It certainly would.

heart's action may not awaken suspicion by its force or irregularity. Nevertheless, its internal lining may be inflamed, and if you listen, the endocardial murmur may convey the momentous fact directly to your ear."

"Now in pericarditis there are the fluid products of inflammation as well as the solid. There is serum as well as lymph. And the signs of fluid effused within the pleura and the pericardium are the same. The fact of its existence, and the measure of its accumulation within the pericardium can only be known by the degree and extent to which the præcordial region, and perhaps some space beyond it, may be dull to percussion. Thus, in pericarditis, dulness sometimes, occupies a part and sometimes the whole of the præcordial region; as high as the second, and even the first left rib; sometimes it extends beneath the whole length of the sternum, except about an inch at the top, and even beneath the cartilage of the ribs on the right side.

"Surely, then, this dulness to percussion is a most important sign, and hardly inferior to, and hardly less diagnostic of, the pathological conditions to which it points, than the exocardial murmur itself.

"But do these two, viz., the murmur and the dulness, bear the same relation to each other as signs of disease within the pericardium, as they have been seen to bear as signs of disease within the pleura? In pleurisy the attrition-sound and the dulness are never coincident, but are always found to supersede each other, one ceasing as soon as the other arises. Is this the case in pericarditis? My own experience would answer almost absolutely 'No!' As soon as I have discovered the exocardial murmur at any part of the præcordial region, so soon have I almost always found dulness to percussion. And, to whatever extent the dulness to percussion has spread beyond the præcordial region, the murmur has accompanied it, even as high as the first left rib, and beneath the sternum, and far beyond it, even to the juncture of the cartilages with the right ribs. Further, I have known dulness of the præcordial region to be the *first* sign, and to subsist several days alone, and yet the attrition sound has been superadded to it, when they have thenceforth continued together. In pericarditis, then, this I take to be the general truth, namely, that the murmur, which is produced by lymph deposited upon the surfaces of the membrane, is neither abated, nor abolished, nor otherwise altered in its character by the serum effused within its cavity. It is not, however, the *universal* truth."

"But of all symptoms, mere pain is the most inconstant and uncertain, whatever be the disease. It is so in pericarditis. It is present in one case and absent in another, strangely and unaccountably. I have known much pain, when the disease has been of little severity, of short duration and of easy cure: and I have known the severest pericarditis pass through all its stages without pain. All other symptoms have been present to mark its reality and its progress: the murmur and the præcordial dulness; and the fluttering heart, and the respiratory anguish. And sometimes the patient has died, and sometimes he has escaped by a tardy and precarious convalescence. But from first to last there has absolutely been no pain.

"Do not be surprised at this. Pleurisy may exist without pain; even acute, rapid, pus-effusing peritonitis. And so too, if in pericarditis there is sometimes no pain, it fortunately happens that there are other signs by which we can fix our diagnosis of the disease equally well without it.

"See what a strange, unequal and uncertain light pain is found to throw upon diagnosis and treatment! We find it where we do not look for it, and look for it where we do not find it. Its presence is no sure proof, its absence is no sure negation of disease.

"But still pain is a most important symptom. Where there is pain, we should always think of disease, always search after disease, and always require strong circumstances to convince us that disease does not exist. Where there is no pain in a part suspected of disease, we should never on that account conclude it to be healthy, and never be content until we find other circumstances to convince us that it is really so."

Lecture eight is devoted to statistical accounts of the frequency of disease of the heart in acute rheumatism, and of the results of treatment, &c., in St. Bartholomew's Hospital.

"Between the years 1836 and 1840, both inclusive, there occurred under my care at St. Bartholomew's Hospital 136 cases of acute rheumatism.

"Of these 136 patients, 75 were males and 61 were females; of the 75 males the heart was affected in 47, and unaffected in 28.

"Of the 47, the seat of disease was the endocardium alone in 30; the pericardium alone in 3; and both the endocardium and pericardium in 7. And, while the heart was undoubtedly affected in seven others, the exact seat of its disease was uncertain.

"Of the whole number of males in whom the heart was thus variously affected, 3 died. And in these 3, the pericardium and the endocardium were both inflamed.

"Of the 61 females, the heart was affected in 43, and unaffected in 18.

"Of the 43, the seat of disease was the endocardium alone in 33; the pericardium alone in 4; and both endocardium and pericardium in 4; and the exact seat of the cardial disease was doubtful in 2.

"Of the whole number of females in whom the heart was thus variously affected, none died.

"The account of males and females, taken together, will stand thus:—

| | |
|---|---|
| Cases of acute rheumatism - - | 136 |
| Heart exempt in - - - - | 46 |
| Heart affected in - - - - | 90 |
| Seat of disease in the heart— | |
| Endocardium alone in - - - | 63 |
| Pericardium alone in - - - | 7 |
| Endocardium and pericardium in - | 11 |
| Doubtful in - - - - - - | 9 |
| Deaths - - - - - - | 3; |

in all of whom both endocardium and pericardium were affected.

"Here are momentous facts, which go, I suspect, a good deal beyond the ordinary notions entertained by medical men of this matter. It is believed that among the sufferers of acute rheumatism, an individual now and then unluckily has his heart inflamed. The thing is looked upon as an accident which, if not very rare, yet is not very common. But it appears, from the event, not of a dozen or twenty cases merely, but of a number large enough to furnish the measure of what naturally belongs to the disease, that as many as two-thirds of those who have acute rheumatism also suffer inflammation of the heart."

Our limits will not allow us to make more copious extracts on these subjects, and we pass to "the treatment of acute rheumatism considered preparatory to the treatment of its accompaniments, endocarditis and pericarditis."

"It has been said that, in the treatment of acute rheumatism, one trusts entirely to venesection and cures it, another to opium and cures it, and another to drastic purgatives and cures it. Here, among several indications which offer themselves to his choice, the physician takes a single one, and makes it the sole mark and scope of his practice, trusting that, when he has effectually attained it, the complex actions and sufferings which constitute the disease will be brought to an end.

"Thus, he takes the high vascular action of acute rheumatism and sees the whole disease represented in it, and is solely intent upon subduing it by venesection, expecting that as he pulls down the circulation, the fever, the nervous disquietude, and the pain, and the swelling, will all cease, and the various secreting organs of the body will resume their natural functions, and that thus the actions of health will gradually supersede the actions of disease. Or he takes the nervous disquietude and the pain of acute rheumatism as the representative of the entire disease, and deals with it accordingly, being solely intent upon moderating them with opium, and expecting that, as they subside, the high vascular action and the fever will subside along with them, and that the secretions will return to their healthy measure and kind. Or he takes the state of the several secretions, their deficient quantity, and their unhealthy quality, as the representative of the entire disease, and so addresses his treatment to those organs whose secretory functions are more immediately within the reach of medicine, the stomach and bowels and liver, and he gives large and repeated doses of calomel, and follows them with large and repeatcd doses of purgative medicine. This he does, and this is all that he does; and having done it effectually for a few days, and obtained very large and bilious evacuations, he expects that the fever, and high vascular action, and nervous disquietude, and pain and swelling will all cease, and the patient will be well.

"Let me repeat my testimony to the success of this practice in acute rheumatism; the practice, namely, of choosing some single indication and steadily pursuing it to its fulfilment. It is a very rational practice. It is founded upon experience and it compasses its end by very simple means; and the manner of its successful operation may be well conceived, if it cannot be entirely explained, in the present state of our knowledge. Disease is a series of new and extraordinary actions. Each link in the series is essential to the integrity of the whole. Let one link be fairly broken, and the integrity is spoiled, and there is an end of the disease; and then the constitution is left to resume its old and accustomed actions, which are the actions of health.

"But, you may ask, is the treatment of acute rheumatism really so plain and simple an affair in all cases? Is there nothing else to be done, but out of several purposes (or indications of treatment, as we call them) to choose judiciously some single one, and pursue it resolutely and effectually by the simplest means? And is this the practice to which the cure of the disease may be safely trusted in all cases? Certainly not—certainly in a small proportion of them only.

"But it is not without reason that I have dwelt upon this practice of single indications and single remedies. For though capable of being strictly followed in a few cases only, it contains a principle of large application, which helps and furthers the treatment of all cases of this disease, and of many diseases besides."

"But there is a plan of treating acute rheumatism which is juster and safer, and applicable to more cases, and more successful than any of them. And that plan is a compound of all three.

"This compound method, while it works with all the means which have been recommended, stops short of what is harsh and excessive in their use, and yet compasses with more certainty the successful result.

"For I believe that, in the treatment of this disease, and in the same cases, by the judicious use of opium you may spare blood, and by the judicious use of bleeding you may spare opium; that by calomel and purgatives properly administered, you may make bleeding and opium less needful, and that by bleeding and opium discreetly employed you may leave the less to be effected by calomel and purgatives.

"Hitherto you have seen how, in the management of acute rheumatism, you may deal with blood-vessels, and with nerves, and with secreting organs, separately, and with what effect. Presently you will see how and with what effect you may deal with them simultaneously; and how your different remedies once set a-foot, and pursuing different paths, meet and end in one purpose—and that purpose the cure.

"'As many arrows, loosed several ways,
Fly to one mark.'"

The treatment of acute rheumatism is continued in lecture eleventh:

"In several cases, however, the proportion of the remedies to each other would vary. Sometimes more, sometimes less, blood-letting would be called for, and often none at all. So too, more or less opium, and more or less of calomel and aperients.

"I have remarked of the effect of blood-letting in acute rheumatism, that it belongs to it rather to render the disease more curable by other remedies than to cure it itself. Blood-letting, therefore, properly, takes the lead of other remedies in point of time. For if it be necessary, the whole treatment must tarry, and other remedies come short of the good of which they are capable until, it is performed. On the first view, then, of the patient, the immediate question is—should he be bled? and if so, to what amount? This question it would be foolish and dangerous to pretend to settle anywhere but in the wards of the hospital, and with the very patient before you, and your finger upon his pulse. Well! and what then? Why! then if you judge there is more force of circulation than calomel and purgatives operating upon the bowels, aided by the soothing effects of opium upon the nervous system, will be able to abate, you may bleed.

"This is the best direction I can give. But be it the best and wisest that can be given, it must be utterly useless, nevertheless, except to those who are or shall be constantly busied about the sick. For, by this direction, whether to bleed or not to bleed is made to wait upon a judgment which must first be formed upon two points. And these two points nothing but the most constant bedside experience can make sure of. They are these—the exact force of the circulation in a particular instance, to be ascertained by the pulse; and the probable power of calomel, and purgatives, and opium to reduce it, to be estimated by the ordinary effects of the same remedies.

"I do not wish to exaggerate the difficulties of medical practice; neither do I wish to conceal them. I am sure you will never surmount them unless you first feel and acknowledge them. And some practical experience is needed even for this.

"When from the pulse I have considered venesection necessary to bring down the circulation, the loss of between twelve and sixteen ounces of blood has generally been enough to answer the purpose in view; and the venesection has seldom been repeated.

"The opium, and calomel, and purgatives I have been accustomed to give in combination thus:—With the calomel administered at night, according to its quantity, I have united more or less of opium. To ten grains of calomel I have added one grain of opium; or to five of calomel I have added half a grain, continuing to give them together in the same proportion, night after night, as long as they are needed. Then on each succeeding day, when a large purgation of the bowels has been duly obtained, I have still given the opium alone, or with saline draughts, in doses of half or one-third of a grain every five or six hours. And thus, with the large quantity at night, and the smaller quantities during the day, about two grains of opium have been commonly taken in the course of twenty-four hours."

The variation in the treatment of the disease when it affects the heart, and the reasons for it, are clearly set forth in the following extract from lecture twelfth:

"When the question is of the joints, it might be laid down as a maxim of practice, to treat the rheumatism (or the general disease) and let the joints take care of themselves. But when the question is of the heart, the maxim might be stated conversely; to treat the heart, and let the rheumatism take care of itself. For inflammation of the heart, whether endocarditis or pericarditis, being the accompaniment of acute rheumatism, is to be managed by the same methods and remedies as would be, were it alone and idiopathic. And these methods and remedies are such as might not be employed at all, or certainly not to the same extent, in a rheumatism attended only by inflammation of the joints.

"Now I admonish you that I am going to enter into bed-side details. For I can teach you nothing unless I do, and you can learn nothing unless you attend to them. Blood-letting and mercury, and opium, are your remedies for these diseases of the heart. And so they are for acute rheumatism irrespective of inflammation of the heart. And so they are for twenty diseases besides. But little practical instruction is conveyed simply by announcing the fact. For in each of the twenty diseases, nay, in each case of any one of them, they may need to be employed in different modes and measures. Thus they are only *conditionally* curative after all. But is not this almost as much as can be said of the application of any remedy to any disease? Conditions mix themselves with all medical practice. To know the disease and to know the right remedy, are only first steps towards the right treatment. The success, and even the safety of practice, come from knowing things which lie far beyond. Stop here, and you will soon find it much easier to kill a man with the *right* remedy than to cure him.

"Bleeding, mercury, opium, the very remedies you used in acute rheumatism, are (I say) still your main reliance, when inflammation attacks the heart; but bleeding in different modes and measures, mercury directed to a totally different purpose, and opium given with more than one single intention.

"As soon as inflammation is known or suspected to have reached the heart, mercury must be given without delay. Or should mercury be already in use, as a remedy for acute rheumatism, with the intent of obtaining large evacuations from the bowels, it must at once have a new direction given to it. The irritation it has produced within the abdomen must by all means be pacified, and its constitutional impression must now alone be thought of—that impression, of which salivation is the best evidence, of which (as far as we know) it alone of all remedial substances, is properly and exclusively capable, and which, under favorable circumstances, is largely counteractive of inflammation."

The next four lectures are devoted to the consideration of mercury as a remedy in rheumatic and other inflammations, and constitute rather too high an eulogy upon its powers. This remedy possesses great virtue in curing inflammatory affections in general, yet we do not subscribe to that free and almost universal application of it here recommended. And although the induction of ptyalism in the treatment of inflammations of the heart, iris, and some other important organs may properly be regarded as a sine qua non, still we should be extremely careful how we produce a disease often so destructive in its tendencies and, even in many inflammatory cases, of doubtful utility.

Lecture seventeenth treats of pericarditis independent of rheumatism. The following will be new to many of our readers:

"There is no structure of the body more liable to inflammation than the pericardium. Of those who have reached adult age and upwards, one half (it appears) have suffered pericarditis at some period of their existence. But then, in the vast majority of cases, it is neither detected, nor perhaps detectible, during life. It comes and goes unnoticed, and neither by itself while it remains, nor by its effects when it has ceased, does it do any amount of injury capable of interfering with the healthy actions of the heart. Hence, in five cases out of six there is no clinical history to be given of pericarditis. How and when, and under what circumstances it takes place in the living man, we have not the smallest experience. All our knowledge of it is from its effects which we discover in the corpse."

It appears to have been shown by Mr. Paget that the white spots on the heart, so commonly observed in dissections, are sequelæ of inflammation. The following is the author's opinion of its importance:

"Doubtles it would be to our credit, that pericarditis in all its slighter degrees should come within our knowledge and treatment. But, because this is not the case, mankind has suffered nothing. For such pericarditis is harmless from beginning to end. It puts life to no present peril, and does no ultimate injury by its effects. Those white spots and slender adhesions of the pericardium are often found where there is not a vestige of disease besides; and then the heart at the same time is so constantly found perfect in size, and form, and capacity, that they may be considered as things almost purely innocent."

In lecture eighteenth it is shown how, after the inflammation has subsided, the patient often sinks from a want of energy in the system to make reparations.

The next four lectures give a most interesting account of the permanent injuries of the heart consequent upon endocarditis and pericarditis, such as adhesions, thickening of the membranes, contractions, and valvular derangements; but as we cannot enter into details sufficiently to make it interesting without exceeding our limits, we pass this subject by.

After speaking of inflammation of the muscular substance of the heart, which is shown occasionally to take place in the acute form; sometimes going on to suppuration, but which is seldom detected until after death, our author dwells upon softening and fatty degenerations; the former of which from the following extract, would seem to be much more common than generally understood, and of great interest to the practitioner:

"I have been looking over M. Louis's admirable book upon fever, and find him laying great stress upon the intermitting and irregular pulse, attesting its formidable import, declaring how few who have it recover, and stating that he has found in almost all of those who have had it and have died, a softening of the heart's muscular structure.

Dr. Stokes, of Dublin, has lately been directing attention to the same morbid condition of the heart among the formidable contingencies of fever. And he had done so, with some novelty in his views of the thing itself, with a more precise notice of its diagnostic signs and (what is most important of all) with the discovery, that these contain indications which may be safely trusted to decide one of the most difficult points of practice in the management of fever. Dr. Stokes holds this softening of the heart to be a proper and special effect of fever, and its diagnostic signs to be the impulse of the ventricle becoming almost or altogether imperceptible, and the systolic sound at the same time almost or altogether inaudible. And he considers that the impulse and the sound together being thus weakened or abolished, whatever in other respects be the patient's condition, call at once for stimulants as his only means of safety: and that his safety is insured as soon as a fairly perceptible impulse and a fairly audible sound are thus restored to the heart. The impulse and the sound thus ceasing and thus returning are, under the circumstances, diagnostic signs as nearly perfect as can well be conceived.

"But there are certain chronic constitutional diseases, in which the blood becomes corrupt in quality or in some essential constituent, in its red globules for instance, as in scurvy or chlorotic anæmia. A pulse deficient in power and intermitting is among the charactistics of such diseases, and when death takes place, the heart is found softened.

"I have something to say practically upon this subject of the softened heart which must be reserved for another place.

"That other condition of the heart, viz., the conversion of its muscular substance into fat, which acquires an importance from the serious results to which it leads, may be described in a few words.

"The healthy heart is always more or less marked upon its surface with streaks of white, and this appearance comes from the deposition of fat in the cellular texture which unites the serous covering with the subjacent muscular structure. It is found chiefly where the venæ cavæ unite to form the the right auricle; also at the base of the ventricles and along the line which marks the boundary between the two, and around the great blood-vessel as they emerge from the heart. But when fat is found in more than these situations and in more than the natural quantity, it is not so much added to the healthy substance of the heart as existing at its expense and detriment, and the muscular structure is that which especially suffers. The muscular fibre is sometimes pale and wasted like that of a paralytic limb.

"Now the predominance of fat in the heart, whether it be superadded to, or intermixed with, its muscular structure, may be said to constitute a form of unsoundness, partaking in some sort, of the character of disease; moreover, like other unsoundness from disease, it naturally leads to unsoundness from disorganization. The fat heart ends by becoming also a dilated and enfeebled heart."

The subject of hypertrophy, atrophy, dilatation, and contraction are next discussed under the general head of disorganizations of the heart.

These alterations are said to be results of former or present disease either of the heart itself or of some organ or organs intimately connected with it. In many cases they originate from acute rheumatism. Sometimes from disease of the arteries, lungs, liver, curvature of the spine, deformity of the chest, &c.

In reference to the treatment of hypertrophy he says:

"By most writers upon diseases of the heart I find its hypertrophy spoken of as curable. Its muscular substance, having acquired even a large increase of bulk, is considered capable of being again brought down to its normal size and normal force of action, by medical treatment. And of the remedies contributing to this result I find venesection represented as the chief. Now it would be unfair to mankind to abridge the hopes and efforts of medical men in all things possible for their benefit; and the cure of hypertrophy of the heart does not look like a thing which is in its nature impossible. But I must confess that, in the whole course of my experience, I never yet met with a single instance in which I was perfectly satisfied that it was cured. This negative experiencc of mine may not be worth much. Yet I have been a good deal in the way of such things; and it does appear rather strange that what others have seen so often I should never have seen at all. Therefore I may be pardoned for suspecting that physicians, affirming not the mere curability of hypertrophy, but its very frequent cure, were under some mistake."

"Impulse of the heart, taken alone, however great and however extensive it may be, is not a sure physical sign of hypertrophy. Hypertrophy, indeed, cannot exist without excess of impulse, but excess of impulse can exist without hypertrophy. When the impulse of the heart is excessive, and at the same time its sounds are obtuse, muffled and indistinct, and the præcordial region presents a larger space than natural which is dull to percussion, then the signs of hypertrophy are complete. And hypertrophy so sure and unquestionable was never cured within my experience. But when the impulse of the heart is in excess, and at the same time its sounds are as loud and clear as ever, or louder and clearer still, and the whole præcordial region is quite resonant to percussion save the small space which is naturally dull, then the signs of hypertrophy are incomplete. Yet if this be enough to constitute hypertrophy, I have seen and treated it successfully in a hundred instances. But in the meantime I have not thought that I had to do with such affection, or ever claimed the least credit for curing it."

In reference to the treatment of atrophy our author gives even less encouragement:

"Atrophy of the heart, or attenuation of its muscular substance, is the form of disease which is naturally opposed to hypertrophy. Now of this atrophy, I doubt whether it can ever be made a distinct object *respective to cure.* I doubt whether the heart, having suffered such loss of substance from special disease of its own, can ever be made to recover it again by help of medicine.

"When, owing to defect of nourishment, from fever, or from disease in particular organs, or from accident, the whole becomes weak and attenuated, the heart may, perhaps, (I am not sure of the fact,) share the general disorder and become weak and attenuated also. And when the whole body, owing to better nourishment, becomes robust and lusty again, the heart may, perhaps, (I am not sure that it does,) recover its natural strength and substance.

"But all this, although it may originally spring from disease, yet, as far as the heart is concerned, has to do, not with disease, but with degrees of health. The full energies of health revive, and the heart is re-invigorated."

Dilatation is either produced by hypertrophy of the walls of the heart or by their attenuation from softening or atrophy, and of course the treatment is included under one of these heads. Hypertrophy and atrophy have been spoken of, and it remains but to treat of softening:

"Again, in fevers, when the skin is dusky, and the impulse and systolic sound of the heart both fail, and death is imminent and threatening, and yet under the seasonable use of wine and stimulants the skin brightens and the heart is again felt and heard, and with its returning impulse and sound all inauspicious symptoms are gradually cleared up and recovery is finally complete, then surely we cannot be wrong in believing, first, that the heart had been softened, and had afterwards recovered its natural texture and power, and secondly, that this recovery of its natural texture and power was mainly instrumental in saving life.

"This softening of the heart in fevers is no new fact. But the knowledge of the precise auscultatory signs which denote its softening, and of the precise auscultatory signs which denote recovery, this indeed is new knowledge, and we owe it to Dr. Stokes, of Dublin. And further, the detection, in these same auscultatory signs, of one precise and plain *indication* to guide us in a most difficult point of medical practice, viz., the administration of stimulants in fever, this too is new, and this too we owe to the same sagacious physician. Whoever discovers a single new indication of treatment, which shall prove just, aud true, and comprehensive, does a better service to mankind than if he found out twenty new remedies.

"From what I have seen I am convinced, that this softening of the heart, by corruption of the blood in fevers, is often a very rapid process, and never a very slow one. And I am also convinced that the recovery of the heart (when it does recover), by restitution of the blood, is often a very rapid process and never a very slow one. The blood is soon changed *from* its healthy state, and soon changed back again *to* it. The process seems more chemical than vital from its mere rapidity.

"Now, as to treatment, it should seem that whatever had power to restore its healthy quality to the blood would be the remedy most suitable to recover the heart from its softened condition; and theoretically one would look to chemistry for it. But chemistry has no such remedy to offer us; and so we are left to make the best of our mere experience, and to sustain the nervous system and the movements of the heart by simple stimulation, while nature transacts the business of reparation in her own way. Our remedies have no other purpose, and probably have no other effect, than to keep up a little life until the blood is able spontaneously to restore itself, and by consequence to restore the heart, and whatever other organs may have suffered the like detriment with it, to the conditions of health.

There is also a softening of the heart which belongs to certain diseases of a chronic kind characterized by an unhealthy state of the blood; such as scurvy and chlorotic anæmia. When people die of scurvy the muscular structure of various organs throughout the body is found loose and soddened, and without its natural cohesion, and of the heart among the rest. And when they die of chlorotic anæmia, internal parts are not only found as bloodless as the surface but also loose of texture. Such is the condition of muscles, and among the rest, of the heart.

"But recovery is infinitely the more frequent event of these diseases; even full and complete recovery, when it is impossible to doubt that the heart, and whatever other parts have suffered detriment, are restored to their natural integrity.

"Now in these cases the softened heart has no special remedy. Its treatment is merged in the treatment of the scurvy or the anæmia. Cure the one by lemon juice and the other by steel, and you cannot help but cure the softened heart."

But softening of the heart, says the author, is generally secret in its beginning and course until, with a train of concomitant affections, it is manifested, and we cannot tell which preceded or followed:

"The patients are in the decline of life, or they have forestalled the season of old age by intemperate habits. Their nervous system may be shattered: their arteries may have undergone extensive changes of structure: their livers may be enlarged, their kidneys may be granulated. All of these forms of disease may be, and some of them are sure to be, conjoined with softening of the heart, before it comes to be known and to be treated.

"And, further, before it comes to be known and to be treated, the softening of the heart is already a part only of a complex disease in the organ itself. The very same tissue, the muscular tissue, has undergone other disorganization, besides softening. Either it is augmented in bulk and hypertrophied, or it is diminished in bulk and attenuated. This chronic softening of the heart is (as far as I know) always united either with hypertrophy or attenuation.

"Further, a heart softened and hypertrophied, or a heart softened and attenuated, has (as far as I know) always one or both of its ventricles dilated.

"Thus, this chronic softening is a part of threefold disorganization of the heart. And, if each part separately offers (as it does) but a slender hope of cure, the complex of the three may be set down as incurable. No bark or steel or tonic remedy can now reach the heart and strengthen it. No mercury or iodine or alterative remedy can now reach the heart and change it to its natural state again."

The work closes by a consideration of the sequelæ of diseases of the heart, which are treated in a masterly manner. Amongst the most frequent of these are affections of the lungs. Why this should be the case, is plain from the intimate relation sustained between them and the heart, and the proximity of these organs to each other.

Our author regards that obscure affection, angina pectoris, as being a spasm of the heart, and gives numerous cases from which deductions are drawn to sustain the opinion.

But as we have already dwelt at considerable length, we will draw our article to a close.

Our object is, while we give a general idea of the character of the work, to furnish from it such facts and conclusions as shall be of service to the physician.

We hope to have the influence of directing the attention of practitioners to the conditions of the heart in disease which are certainly too often neglected. E.

